# Retroperitoneal Knee Pain: An Unusual Case Report and Review of an Ancient Schwannoma

**DOI:** 10.7759/cureus.2216

**Published:** 2018-02-21

**Authors:** Ibrahim M Hanif, Nilesh H Pawar, Wing Yan Mok, Melvin Chua

**Affiliations:** 1 Department of General Medicine, Sengkang General Hospital, Sengkang Health, Singhealth, Singapore; 2 Department of Radiology, Sengkang General Hospital, Sengkang Health, Singhealth, Singapore

**Keywords:** ancient schwannoma, nerve sheath tumor, retroperitoneal knee pain, schwannoma, abdominal mass, tinel's sign

## Abstract

Schwannomas are nerve sheath tumors that occur in Schwann cells. They are usually benign, but malignant transformation can occur. Symptomatology depends on the involvement of the surrounding tissues or the mass effect of the tumor. We describe a case of a 28-year-old man who initially presented with right iliac fossa pain associated with radiating pain over the anterior and lateral aspect of his right knee. Following subsequent investigations, we found a retroperitoneal schwannoma of the right lateral femoral cutaneous nerve. The key to our diagnosis was the referred pain to his right knee, which gave us a clue of possible neuropathic pain. Our patient highlights the need to consider a unified diagnosis when faced with an incongruent set of symptoms. Magnetic resonance imaging is the diagnostic modality of choice for the diagnosis of schwannomas. Treatment is directed towards symptomatic control. Surgery, radiation, and, in rare instances, chemotherapy are the major treatment modalities employed.

## Introduction

Schwannomas are rare, benign, neurogenic tumors of Schwann cells (i.e., cells forming the outer insulating layer of the peripheral nerves). They comprise about 5% of all benign soft tissue tumors and are usually located in the head and neck regions and upper and lower extremities [1]. Malignant transformation can occur very infrequently. Ancient schwannoma is a rare subtype characterized by degeneration and hypocellular areas. The retroperitoneal location is an unusual site for schwannomas, accounting for about 0.3% to 3.2% of all schwannomas [2]. The most characteristic features were a Tinel’s-like sign [3] and a slowly progressive natural history between the onset of symptoms and surgery, typically several years.

We describe a patient with a retroperitoneal schwannoma whose initial presentation was right iliac fossa pain associated with a referred pain to the lateral aspect of his right knee. Following subsequent investigations, we found a retroperitoneal schwannoma of the right lateral femoral cutaneous nerve causing pain over the lateral side of his right knee.

## Case presentation

A 28-year-old man presented to the emergency department with right iliac fossa pain, which had been present for one week and worsened over the last day. On examination, he was afebrile, blood pressure was 115/65 mm Hg, heart rate was 89 beats per min, and oxygen saturation was 98% at ambient air. His abdomen was soft with a palpable 5-cm mass in the right lumbar region. Palpation of the mass elicited pain over the lateral aspect of his right knee. Results of an initial laboratory evaluation (Table 1) were normal as well.

**Table 1:** Laboratory Investigations

Test	Result	Reference range
Urea (mmol/L)	4.0	2.7 - 6.9
Creatinine (µmol/L)	71	54 - 101
Sodium (mmol/L)	137	138 - 146
Potassium (mmol/L)	3.8	3.6 - 5.0
Random blood glucose (mmol/L)	4.8	3.9 - 11.0
Albumin (G/L)	45	40 - 51
Alanine transaminase (U/L)	30	6 - 66
Aspartate transaminase (U/L)	31	12 - 42
Amylase (U/L)	61	38 - 149
Haemoglobin (g/DL)	15.4	14 - 18
White blood cell count (10­­^9^/L)	7.76	4 - 10
Platelet count (x10­­^9^/L)	190	140 - 440

**Figure 1:**
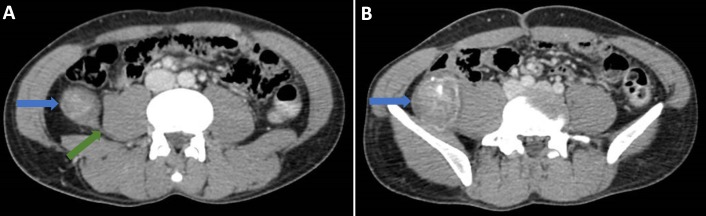
Computed tomography scan of the abdomen and pelvis in portal venous phase (A and B). A) The axial slices show a well-defined mass (blue arrows) in the right iliac fossa, which is separate from the adjacent large bowel and has a tail (green arrow) superiorly and inferiorly (see Figure 2). The mass itself shows heterogeneous enhancement with some coarse calcifications. It is located along the course of the lateral cutaneous nerve. The overall appearances are suggestive of a nerve sheath tumor. Figure 1B shows the mass with heterogeneous enhancement and calcifications (blue arrow). A soft tissue tumor, such as fibrosarcoma, was thought to be a less likely differential diagnosis at this stage.

**Figure 2:**
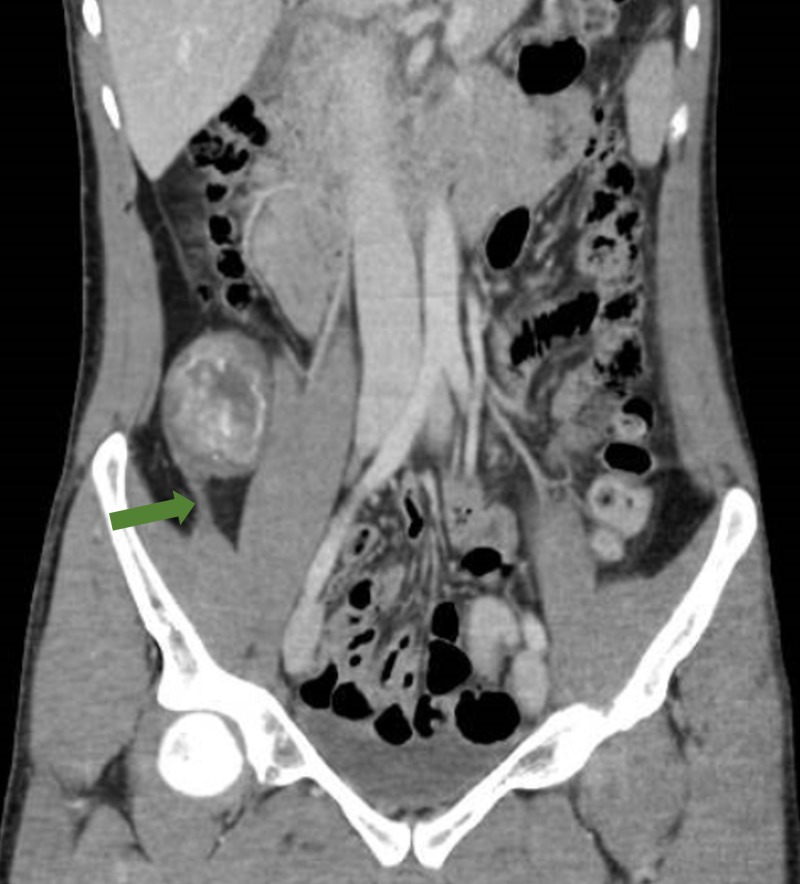
Computed tomography of the abdomen and pelvis in portal venous phase (coronal reconstruction). The inferior tail of the lesion (green arrow) is visible on this reconstruction.

**Figure 3:**
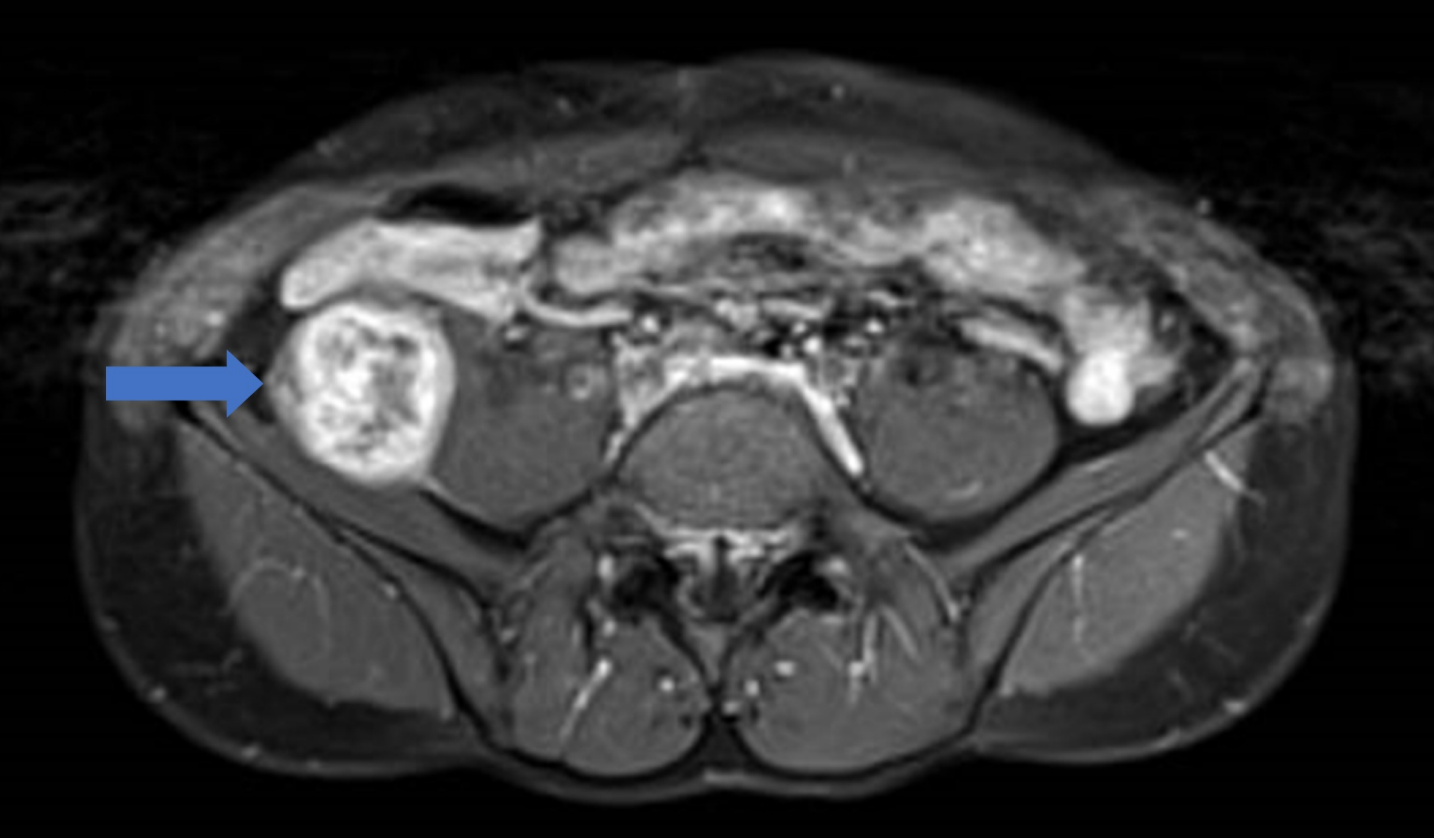
Magnetic resonance image (MRI) of the abdomen with contrast agent. The image is a T1-weighted fat saturation sequence with contrast. It shows the same well-defined lesion in the right iliac fossa with bright heterogeneous enhancement, as well as areas of calcification (blue arrow). The nerve leading to the lesion is much better depicted on MRI. The lesion follows the route of the right L3 nerve root with its distal tail appearing to be communicating with the lateral femoral cutaneous nerve. There was no adjacent fat stranding or edema; the overall appearance would favor a schwannoma over a fibrosarcoma.

An abdominal radiograph performed on admission did not reveal any abnormality. A computed tomography (CT) scan of his abdomen (Figure 1A, 1B, Figure 2) was done, given the palpable mass; the CT revealed an ovoid heterogeneous mass with intralesional enhancement and calcification in the right iliac fossa. It appeared to have a tail superior-posteriorly and was located along the course of the right lateral femoral cutaneous nerve, raising the possibility of a schwannoma. Magnetic resonance imaging (MRI) of the abdomen was done for further evaluation and revealed an ovoid mass measuring approximately 4.6 cm x 4.2 cm x 5.7 cm at the level of L3 - L4 with a tail-like appendage to the proximal and distal parts with features highly suggestive of an ancient schwannoma (Figure 3). A CT-guided biopsy was performed, which confirmed the diagnosis of schwannoma. The histopathology of the mass revealed the schwannoma to be benign. The mass showed cores of spindle cell lesions with moderate cellularity. The spindle cells were bland-looking and arranged in vague fascicles, set in a collagenous stroma. They exhibited wavy nuclei with focal nuclear palisading. No mitosis was seen. Tumor cells were diffusely and strongly positive for S100, while being negative for CD117 and DOG1.

Pain over the right knee was due to nerve compression. Treatment options were discussed with the patient. The patient was not keen for surgical resection of the tumor due to financial problems and as the surgical resection could possibly result in residual numbness over the leg and may affect his gait. Therefore, we planned to manage the patient conservatively with analgesia. His pain was controlled well with gabapentin, 300 mg every night.

## Discussion

Schwannomas are often an incidental finding, presenting as neuropathy due to the affected nerve or with vague, nonspecific symptoms attributable to their mass effect on adjacent structures. Tenderness and numbness are frequently reported in cases of ancient schwannomas [3]. Our patient had a schwannoma on the lateral femoral cutaneous nerve and presented with a rather characteristic radiating pain upon palpation of the tumor (i.e., a Tinel’s-like sign), which gave us a clue to the diagnosis prior to imaging. The anterior branch of lateral femoral cutaneous nerve innervates the anterolateral aspect of the thigh [4]. The lateral femoral cutaneous nerve is formed from the posterior divisions of the L2 and L3 roots and further divides into anterior and posterior branches [4].

Primary retroperitoneal tumors are indeed rare and about 80% to 90% are malignant, which often involves sarcoma. Schwannomas, which are generally benign, account for about 0.5% to 3% of tumors in the retroperitoneum, and they tend to occur in the paravertebral and presacral areas or near the kidneys [2]. When evaluating a retroperitoneal tumor, the paucity of serious clinical signs and symptoms, despite the relatively large size of the tumor, may point towards a benign schwannoma.

Histologically, typical schwannomas are composed of intermixed Antoni A components (i.e., very cellular area consisting of Schwann cell nuclei) and Antoni B areas (i.e., less cellular with myxomatous and cystic changes). Ancient schwannomas are characterized by degenerative changes mainly seen as calcifications, areas of cystic necrosis and hemorrhage, and with a predominance of Antoni type B architecture rather than Antoni A. The degenerative changes seen in ancient schwannomas are reflective of its slow-growing nature.

Both CT scans and MRIs can be used to detect retroperitoneal schwannomas, but MRIs are superior for tissue component analysis of the tumor [2]. The radiological features of schwannomas are similar to those for ancient schwannomas, and below are some radiological clues which may indicate a retroperitoneal schwannoma despite its rarity:

1. They can generally grow to a relatively large size (10 cm to 20 cm) with minimal symptoms due to their slow-growing nature and the non-constricting space of the retroperitoneum [1].

2. They are solitary ovoid or spherical, well-circumscribed, and have smooth borders, which usually do not invade or obstruct surrounding structures [1].

3. The CT appearance is that of a heterogeneous tumor reflecting the varied histologic components of Antoni type A and B cells and cystic degenerations [1].

4. The cystic changes seen in benign schwannomas do not distinguish them from retroperitoneal malignancies, which are often heterogeneous as well, but the smooth, regular border of schwannomas (due to the fibrous capsule) allows them to be recognized for their benign nature [1].

5. In MRI, the tumors are isointense on T1-weighted images relative to the surrounding muscle and hyperintense on T2-weighted images. The intensity of the signal in T2 is inversely proportional to the cellularity of the tumor, as opposed to the noncellular and necrotic zones that accentuate the T2 hypersignal. Schwannomas are strongly enhanced by gadolinium contrast, and this will involve the tissue components, as well as the septa and walls of the tumor [2].

Thus, MRI is the diagnostic modality of choice. Malignant schwannomas have a poor prognosis, whereas benign schwannomas have an excellent prognosis. Although treatment is generally directed towards symptom control, surgery, radiation, and, in rare instances, chemotherapy are also employed depending on the clinical context and patient's preference.

## Conclusions

Retroperitoneal schwannomas are very rare. They are usually benign lesions, although malignant transformation can rarely occur. MRI imaging can provide certain important clues which point towards the diagnosis of a schwannoma. These imaging clues, when coupled with a congruent clinical picture and symptomology, can aid in the diagnosis even before the histological confirmation. Treatment is usually directed towards symptom control and surgical treatment may be favored depending on the clinical setting.

## References

[1] Hughes MJ, Thomas JM, Fisher C, Moskovic EC (2005). Imaging features of retroperitoneal and pelvic schwannomas. Clin Radiol.

[2] Hoarau N, Slim K, Da Ines D (2013). CT and MR imaging of retroperitoneal schwannoma. Diagn Interv Imaging.

[3] Isobe K, Shimizu T, Akahane T, Kato H (2004). Imaging of ancient schwannoma. AJR Am J Roentgenol.

[4] Anloague PA, Huijbregts P (2009). Anatomical variations of the lumbar plexus: a descriptive anatomy study with proposed clinical implications. J Man Manip Ther.

